# Surgical management of renal tuberculosis

**DOI:** 10.4103/0970-1591.42620

**Published:** 2008

**Authors:** Sriram Krishnamoorthy, Ganesh Gopalakrishnan

**Affiliations:** Department of Urology, Christian Medical College, Vellore, India

**Keywords:** Hydronephrosis, nephrectomy, renal tuberculosis

## Abstract

Tuberculosis (TB) is one of the major health problems that our country is facing today. Despite active interventions by our government, control of TB still remains to be achieved. The emergence and exponential growth of the human immunodeficiency virus and drug-resistant strains threaten to further complicate the TB situation in our country. Even in this era of advanced chemotherapy, many lives are lost every day in our country. Tuberculosis of the urinary tract, despite being one of the commonest forms of extra-pulmonary TB, is generally overlooked. Most patients present with vague lower urinary symptoms typical of urinary tract infection. In this article, we shall highlight the various issues related to the surgical management of renal and ureteral tuberculosis.

## INTRODUCTION

Tuberculosis (TB) remains one of the major health problems in our country. About 1.8 million new cases of TB are detected every year in our country, of which one-fifth are extra-pulmonary.[1] Even in the present era of modern chemotherapy and advanced diagnostic modalities, more than 1000 lives are lost due to TB every day in India.[1] Urinary TB is generally suspected only when the symptoms do not improve with usual anti-bacterial agents. In this article, we shall discuss the surgical management of renal tuberculosis.

Renal TB is the most common site of extra-pulmonary TB and comprises 15-20% of all extra-pulmonary tuberculosis.[2] Our therapeutic concepts concerning the disease have changed dramatically, particularly since the introduction of Streptomycin. The cornerstone in the management is multi-drug treatment, with an aim to decrease the duration of therapy and to decrease the likelihood of drug-resistant organisms. With the advent of multi-modal chemotherapy, mortality has reduced considerably. The medical management of renal TB is discussed in detail in the previous articles of this issue. In this chapter, we shall discuss the various urological issues related to the management of renal tuberculosis.

Renal TB, in spite of being a common condition, is highly complex to manage, in view of its various modes of presentation. The diagnosis is often difficult and delayed as TB has myriad clinical and radiological manifestations. Hence, it is necessary to have a high index of suspicion, as this would enable an earlier diagnosis and timely initiation of appropriate management, thereby reducing the morbidity.

## PATHOLOGICAL CONSIDERATIONS

Renal TB is usually sequelae of pulmonary TB that had occurred at least 10-15 years earlier. The bacilli usually are lodged in the cortico-medullary region and form cortical granulomas. These granulomas remain dormant for many years. When the individual's immunity is threatened, there is a reactivation of these dormant bacilli resulting in spread into the medulla, causing papillitis.

The disease process is very slow but as it progresses, it results in extensive necrosis of the renal papillae and may lead to the formation of frank cavities with abscess formation, ultimately resulting in total destruction of the renal parenchyma.

As the disease advances, there may be scarring of the renal cortex resulting in infundibular and pelvi-ureteric junction strictures. The disease may spread into the collecting system, resulting in bacilluria.

It is important for us to know that bacilluria occurs later in the disease process and one must realize that the absence of bacilluria does not exclude the disease.

The result is a non-functioning kidney with extensive calcification involving the entire kidney. There are two mechanisms by which TB can cause renal failure: first, an intrinsic infection within the renal parenchyma, causing obliterative endarteritis and renal impairment with extensive dystrophic calcification involving the renal parenchyma and second, by post-obstructive atrophy secondary to multiple infundibular stenoses or ureteric strictures.[3,4] Ureteral involvement in urinary TB is usually secondary to renal involvement. This usually occurs because of seeding of the ureter from the infected kidney.

## ROLE OF IMAGING

Intravenous urography (IVU) is the hallmark radiological investigation to make a diagnosis of urinary tuberculosis. Computed tomography (CT) urography is now being increasingly used as it provides definite anatomic details of the pathology. Poor bowel preparation is not a factor in interpreting the findings accurately. Moreover, CT also helps to identify associated ureteric lesions and calcifications more accurately. Nevertheless, it would be unfair to discard IVU at this stage. Intravenous urography is still the cheaper investigation and will continue to be used by most urologists. One must also remember that in the follow-up of patients who have undergone reconstructive surgery, IVU will be commonly used because the radiation exposure is higher with CT scan and this would make a significant difference in the women of childbearing age.

**Figure 1:**
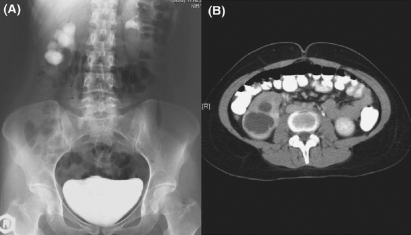
(A) IVU showing a dilated lower calyx. (B) CT scan showing a cavity in the lower pole, non-communicating with the rest of the collecting system

CT is particularly useful in patients with renal parenchymal masses and scarring, thickened ureters and in those who have extra-urinary tubercular manifestations. It has better ability than IVU to reveal the anatomical details of a small urinary lesion as well as extra-urinary changes. Morphological changes of the disease are visible on axial scanning. It also provides information on the functional status of the affected kidney. In addition, if the system is non-functioning, no information regarding the anatomical details will be obtained in IVU, whereas CT will give us a complete picture of the anatomical details of the affected kidney.[5] Figure 1A shows a dilated lower calyx of the right kidney in IVU, but CT clearly shows a cavitation of the lower pole, which is cut off from rest of the collecting system [Figure 1B]. Renal and peri-renal abscesses are seen as low attenuation collections (10-40 HU). If calcification is present, it is more suggestive of tuberculosis.[6] CT is also an excellent substitute for retrograde pyelogram, because in many situations due to an inflamed or contracted bladder, the ureteric orifice could not be identified. Additionally, presence of a golf hole ureter in itself is not suggestive of vesico-ureteric reflux, as in TB, golf hole orifice can be seen concomitantly with obstruction.

**Figure 2:**
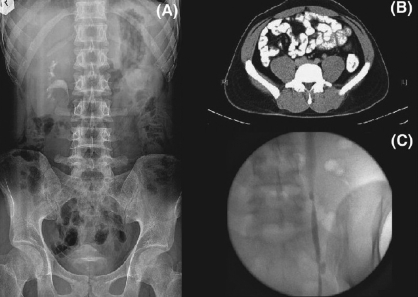
(A) IVU showing a poorly visualized left renal pelvis and a non-visualized left ureter. (B) CT scan showing a left ureteric stricture, which was no recognized in the IVU. (C) Retrograde ureterogram showing multiple upper and mid-ureteric strctures

Many a time, there may be multiple ureteric strictures, which are likely to be missed in IVU, especially if the contrast does not drain across the pelvi-ureteric junction [Figure 2A]. In such cases, CT scan will provide details regarding the distal ureter [Figure 2B]. Before planning any form of reconstructive procedures, it is imperative to do a cystoscopy and retrograde assessment of the distal ureter as well as the bladder, as this would help in planning the most appropriate management [Figure 2C]. It should be remembered that correction of the distal pathology is mandatory before attempting any form of reconstructive procedure of the upper tract.[7]

## EVOLUTION OF ADVANCEMENTS IN THE MANAGEMENT OF RENAL TUBERCULOSIS

Despite the effectiveness of modern chemotherapy, tuberculous infections of the genitourinary tract remain a serious threat to the life of the patient, if the disease is not promptly diagnosed and treated appropriately. From the time Cohnheim presented his “Elimination theory” in 1879,[4] followed three years later by the isolation of the tubercle bacilli by Robert Koch, to the time of therapeutic standpoint with the invention of cystoscope by Nitze, there has been a tremendous improvement in the management of genitourinary tuberculosis.[8]

There has been a paradigm shift in the management concepts in renal tuberculosis. In 1945, Nesbit and associates had reported a 97% nephrectomy rate in their study on 260 patients with urinary tuberculosis.[9] This was an era, when anti-tubercular treatment was not very much available in clinical practice. The treatment concepts shifted towards conservative management, when Bloom *et al.* in 1970, reported that all their 25 patients with non-functioning tuberculous kidneys were conservatively managed and none of them required any form of surgical intervention.[10] Nowadays, with the advancements in chemotherapy and diagnostic modalities, more and more of these patients are diagnosed earlier and treated conservatively. With improvements in surgical techniques, there is a tilt in the management protocol towards reconstructive surgeries of the kidneys and ureters.

By the 1980s, with the increasing availability of anti-TB chemotherapy and with the implementation of the Tuberculosis Control Programme, the incidence and prevalence of TB the world over showed a downward trend. In the late 1990s, the changing patterns of the population emigration and the development of large numbers of immunocompromised and end-stage renal tuberculous disease resulted in a reversal of the downward trend of tuberculosis.[11]

It is very common to observe a patient presenting with storage Lower urinary tract symptoms (LUTS). On further evaluation, he or she may be found to have the characteristic radiological findings in the pelvi-calyceal system, suggestive of tuberculosis. Tuberculosis is a great imitator of other diseases and can present as vague symptoms.

In a patient with unexplained LUTS, urinary T B should be ruled out, as Chang had said, “*The kidney is an inarticulate organ; its vocal cords are the bladder*”.[12] These patients should be managed based on clinical and radiological findings. All patients with active or latent urinary TB require treatment with anti-TB chemotherapy.

## MANAGEMENT OF RENAL TUBERCULOSIS

The invasive or operative procedures for renal and ureteral TB can be categorized into the following groups: (1) drainage for hydronephrosis (ureteric stenting or percutaneous nephrostomy); (2) drainage of abscesses or localized collections; (3) definitive local treatment of the affected part of the kidney (cavernotomy/partial nephrectomy); (4) nephrectomy of the non-functioning tuberculous kidney (open/laparoscopic/retro-peritoneoscopic techniques) and (5) reconstruction of the upper urinary tract (uretero-calycostomy, ureteric reimplantation, ileal ureteric replacement).

**Double-J (DJ) stenting**: It is common in tuberculous kidneys to observe hydronephrosis secondary to stricture involving the pelvi-ureteric junction or the ureter. As these lesions heal by fibrosis, further worsening of the stricture can occur with treatment. A DJ stenting of the affected system will help in restoration of the patency of the drainage system. It facilitates a passive dilatation of the ureter and prevents any further worsening by acting as a splint across the site of stricture. The strictures should be monitored with CT or IVU. If there is deterioration or no improvement after a six-week period, then surgical reimplantation or other minimally invasive procedures including balloon dilatation may be necessary.[13]

**Figure 3:**
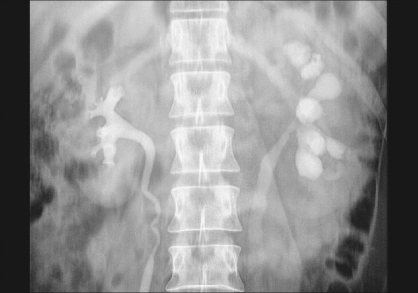
IVU showing multiple infundibular stenosis of the left kidney

**Figure 4:**
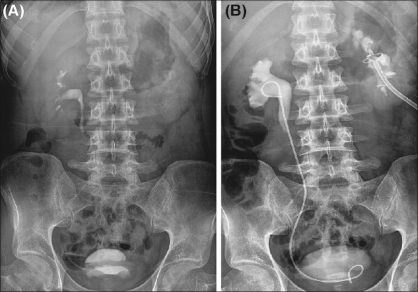
(A) IVU showing a non-visualized left kidney. (B)IVU showing a much improved renal function after percutaneous nephrostomy

**Percutaneous nephrostomy**: Many patients have multiple infundibular stenoses with individual calyces being dilated and non-communicating with each other. There may be a further deterioration of the renal function with chemotherapy, as the calyx may be cut off from the rest of the collecting system secondary to fibrosis. DJ stenting may not be effective in such circumstances, as the individual calyces do not communicate with each other [Figure 3]. In such instances, a percutaneous nephrostomy, if required multiple, may be necessary, in order to protect the functional status of the kidneys [Figures 4A, 4B].[14]

**Cavernotomy**: Sometimes the lesions are characterized by extensive cavitations, which usually occur at the poles of the kidney. The involved calyces do not excrete contrast medium, making it difficult to make a diagnosis of the condition. It is extremely important to have an index of suspicion in these patients, as large conglomerated, non-communicating tuberculoma formed because of infundibular stenosis may radiologically mimic a renal tumor. Ultrasound or CT abdomen usually helps us diagnose the condition.

These patients need to be started on antituberculous therapy and closely followed up. In view of ischemia to that particular segment of the kidney, the drugs may not reach the desired therapeutic levels, resulting in a persistence of the lesion even after the completion of the course of chemotherapy.[15]

When patients do not respond to medical therapy or if the disease process has gone unnoticed, the cavity containing urine may be secondarily infected to form an abscess. Previously, cavernotomy was considered an ideal option. With the advent of Pyrazinamide, which acts intracellularly, and with the availability of better imaging modalities, this procedure hardly has any place in the modern era of management.[16]

**Percutaneous drainage**: sometimes the disease may result in multiple infundibular stenoses or a scarred and cicatrized renal pelvis. In such instances, a percutaneous drainage of the cavity under ultrasound or CT guidance is recommended, along with continuation of the antituberculous therapy. This is a satisfactory method of initial treatment and obviates the need for surgery. It also allows the contents to be cultured for viable organisms. Once the disease process has stabilized, one should plan a definitive procedure after two months of intensive phase of chemotherapy.

**Partial nephrectomy**: Caution should be exercised if there is a calcification in the wall, as slow but insidious extension of the calcification might ultimately destroy the whole kidney. Once this process starts, it is advisable to consider partial nephrectomy.[17] There are only two indications for partial nephrectomy: (1) a localized polar lesion containing calcification that has failed to respond after six weeks of intensive chemotherapy; (2) an area of calcification that is slowly increasing in size and threatening to gradually destroy the entire kidney.

**Figure 5:**
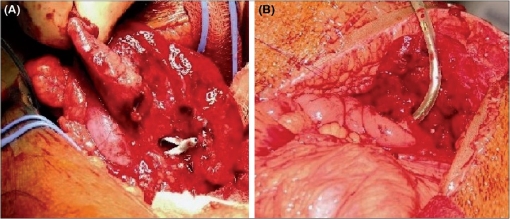
(A) Intraoperative photograph showing the lateral nephrotomy exposing the lower calyceal infundibulum with PCN *in situ*. (B) IIem being anastomosed to the lower calyx

**Calycorrhaphy/infundibuloplasty**: It is also recommended to inject some amount of contrast medium into the cavity and take serial tomograms to assess whether there is any communication with the rest of the collecting system. The non-communication of the cavity with the rest of the system necessitates a closer follow-up and if the calyx is functional may require a calycorrhaphy or infundibuloplasty. If the corresponding pole of the kidney is non-functional, polar nephrectomy is advocated. In patients with a cicatrized renal pelvis and ureter, it may be necessary to perform a lateral nephrotomy to open the infundibulum of all the major calyces, replace the ureter with ileum, and anastomose the proximal end of the ileum to the lateral border of the cavity [Figures 5A, B].

**Nephrectomy**: Sometimes a hydronephrotic kidney may be calcified and non-functional. Appearance of calcification in a tuberculous kidney is a sign of advanced stage of the disease and is reported in up to 50% of patients with renal tuberculosis.[18] When a tuberculous lesion has advanced to the collecting tubules or the renal pelvis, it never heals completely because of a hindered drainage and backpressure effects. When complete healing occurs with antituberculous therapy, the tuberculous process usually is limited to the cortex and has never extended to a pyramid or collecting tubules.[19]

Traditionally, nephrectomy is strongly indicated in patients with a non-functioning tuberculous kidney with calcification or extensive disease involving the whole kidney with other complications including hypertension or coexisting renal cell carcinoma. The reasons being, despite sterile urine after chemotherapy, 50% of the histological preparations of nephrectomy tissues still show active TB. Hence, for all non-functioning tuberculous kidneys, nephrectomy was indicated.[20] On the other hand, Wechsler and associates reported that if the patient is asymptomatic and the disease is only incidentally diagnosed, it is not mandatory to advocate nephrectomy in all patients.[21] This is in contrast to the previous recommendations made by Kerr *et al*. in 1969, who advised nephrectomy for all the diseased or non-functional kidneys. Gupta *et al*. reported an incidence of 33% of the patients with TB requiring nephrectomy.[22] The indications for nephrectomy in a non-functional asymptomatic tuberculous kidney are still debatable, but we feel that any non-functioning tuberculous kidney should ideally be removed. As there could be a possibility of reactivation later in life, it is always better to get rid of the diseased kidney, especially if it is non-functional.

One must remember certain fundamental principles while doing nephrectomy for a non-functioning tuberculous kidney. It is preferable to approach the kidney from behind, as the kidney is more often adherent to the colon anteriorly in view of severe inflammation. It is not necessary to remove the ureter completely as the goal should be to remove as much of the diseased ureter as possible. Every effort should be taken to ligate the artery and the vein separately, in order to prevent the possibility of development of arterio-venous fistula if both are tied together.[16]

Traditionally, tuberculous kidneys were considered a relative contraindication for laparoscopic nephrectomy in view of dense perinephric adhesions, resulting in a higher rate of intraoperative complications and need for conversion to open nephrectomy. The earliest reports of laparoscopic nephrectomy were by Gupta *et al*. where they described tuberculous kidneys to be a relative contraindication in view of high conversion rate.[23] Rassweiler *et al*. suggested that tuberculous kidneys have a very high conversion rate.[24] With improvements in techniques and surgical expertise, more data are available on successful laparoscopic and retroperitoneoscopic nephrectomies.[25–28]

Before exploring a non-functioning tuberculous kidney, it is important for the urologist to consider certain issues.

- Is there a danger of viable tubercle bacilli in a tuberculous kidney?
  Gow *et al*. reported that tuberculous lesions in auto-nephrectomized kidneys did not heal completely and were a potential source of possible viable tubercle bacilli for further infection.[18] However, Bloom *et al*. challenged this concept, when they reported that they could not grow any tubercle bacilli from the caseous material in non-functioning tuberculous kidneys.[10] Wyrens, while analyzing the indications for extirpative surgical procedures for renal TB, concluded that it was possible that organisms were present within the auto-nephrectomized kidney, but were unable to undergo proliferation.[29]
- Is salvage of a poorly functioning tuberculous kidney always possible?
  Gocke *et al*. in a retrospective analysis of 174 patients over 20 years, reported that all 17 patients with renal TB, who required surgical intervention ended up with nephrectomy.[11] Whenever any patient presents with obstruction, the question of salvageability often arises. Ramanathan *et al*. in their study on 82 patients over seven years, assessed the effects of the relief of obstruction on ultimate renal function of the affected kidney and identified certain predictive factors of functional recovery. Patients with a distal ureteric involvement, a good cortical thickness of >10 mm, a GFR of >15 ml/min seemed to have a good potential for recovery of renal function after intervention.[30]

Ideally, percutaneous nephrostomy is the best method to assess the recoverability of renal function. It promptly decompresses the obstructed kidney and helps to estimate the creatinine clearance of the diverted urine. The limitations are that it is an invasive procedure and if the kidney is non-functioning, nephrectomy has to be done in order to prevent the occurrence of a nephro-cutaneous fistula. The other option is to do an internal stenting, which is often difficult as most patients have an associated bladder involvement.

On the other hand, if the patient's GFR is <15 ml/min and the cortical thickness is <5 mm, associated with more proximal lesions, it is a more likely that the kidney is not worth salvaging. In such instances, it is better not to intervene, as these kidneys are less likely to improve after intervention.[30]

## TUBERCULOSIS AND RENAL FAILURE

Tuberculous infection is 6-16 times much more frequent in patients undergoing hemodialysis than in the general population.[31] Even though reactivation results from old caseous foci present in the lungs and lymph nodes, the genitourinary tract is also one of the potential sources for reactivation. Renal disease can be particularly occult and insidious, leading to progressive destruction and calcification of the parenchyma. Renal TB can result in end-stage renal disease by two mechanisms. First, it can inflict direct insult to renal parenchyma by causing obliterative endarteritis of the intra-renal segmental vessels or secondary renal amyloidosis.[32] Secondly, by the obstruction of pelvi-ureteric junction or multiple infundibular stenoses, it can result in obstructive uropathy.[3]

The overall incidence of renal failure reported in the literature is 24%. Gupta *et al*. had reported an incidence of 22.4% in their retrospective analysis of 241 patients.[22]

Renal TB can also result in certain biochemical abnormalities in patients with end-stage renal disease. Hypercalcemia is one such important biochemical abnormality seen in such patients. This usually occurs secondary to abnormal calcitriol production by the granulomatous tissue. This ectopic production of calcitriol by the granulomas is either unregulated or is regulated by ways different from those controlling the normal renal production of this hormone.[33] Peces *et al*. reported that a successful management of TB resulted in resolution of hypercalcemia and an adequate parathyroid hormone response.[34] Any attempts at correction of hypercalcemia alone may prove detrimental to these patients.

We know that renal TB can result in chronic renal failure necessitating hemodialysis and renal transplantation. On the other hand, patients with bilateral renal TB can present with acute renal failure. Gupta *et al*. reported the first incidence of bilateral genitourinary TB presenting with acute renal failure, who was initially managed with preliminary high diversion followed by bilateral replacement of ureters with ileal loops.[35] Conte *et al*. in their report suggested that any patient with acute renal failure in whom no definite cause could be identified, TB should be considered.[36] In patients with renal failure requiring a bowel interposition, a short ileal conduit is preferred over an augmentation of the bladder. Bowel interposition leads to various metabolic abnormalities and already existing renal failure could further worsen with time. Such patients need to be closely followed up as they could develop chronic renal failure necessitating dialysis and renal transplantation.

If the patient presents with obstructive uropathy, it is necessary to do an initial diversion in the form of percutaneous nephrostomy and allow sufficient time for the serum creatinine levels to reach a nadir value, before planning any form of definitive intervention.

## CONCLUSIONS

In view of the global emergency of TB, the WHO “Stop TB” campaign has called for universal adoption of directly observed short-course therapy (DOTS). Even in countries like the United States, a wake-up call is being issued to maintain their national commitment towards elimination of tuberculosis.[37]

Our aim should be to not only control the infection, but also eliminate the disease from the community. Apart from the involvement of the local health departments, the role of clinicians in this endeavor will be to remain vigilant for cases of active disease. The clinicians need to intensify their efforts to identify and treat latent TB infection. Failure to do so will result in loss of hard-won progress towards control of TB and might lead to resurgence of the disease.

Robert Day, in 1932, while analyzing why clinically established TB never completely heals, raised the following queries: being of hematogenous origin, why is the disease bilateral in only one-third of the cases? Are bacilli in sufficient numbers carried by chance to only one kidney? On the other hand, do the organisms reach both kidneys, but gain a foothold in one only?[19] These queries still remain an unresolved enigma.
